# Misclassification and Bias in Military Studies of Mefloquine

**DOI:** 10.4269/ajtmh.17-0246

**Published:** 2017-07-12

**Authors:** Remington Lee Nevin

**Affiliations:** Department of Environmental Health and Engineering Johns Hopkins Bloomberg School of Public Health Baltimore, Maryland E-mail: rnevin@jhu.edu

Dear Sir:

I read with interest the recent analysis by Eick-Cost and others, which examined associations among U.S. military personnel between various neurologic and psychiatric diagnoses and earlier use of mefloquine.^1^ Although not accounting for multiple hypothesis testing, the authors’ finding in subgroup analysis of a significantly increased risk of posttraumatic stress disorder (PTSD) with use of mefloquine is intriguing and calls for further investigation.

The U.S. Food and Drug Administration has warned that neuropsychiatric adverse effects from mefloquine, unlike those from other antimalarials, may last years after use or even be permanent. These lasting effects, which may include vivid nightmares, disordered sleep, anxiety, irritability, anger, cognitive dysfunction, and dissociation, may mimic certain symptoms of PTSD.^2^ Recently, several cases have been reported in which these symptoms have been misattributed among U.S. military personnel to PTSD.^2,3^ Potentially consistent with such adverse effects from mefloquine being more broadly misattributed to PTSD, among a nondeployed subgroup the Eick-Cost analysis found a nearly doubled risk of the diagnosis among those prescribed mefloquine as compared with those prescribed atovaquone-proguanil, after adjusting for common confounders.

Although a significant association of PTSD diagnosis with mefloquine was not seen in comparison to doxycycline, this may reflect the effects of differential exposure misclassification and selection bias. Many of the subjects in the doxycycline cohort were deployed to combat in Afghanistan, during a time when policy changes beginning in 2009 made doxycycline the preferred antimalarial,^4^ and when drug use was increasingly subject to electronic documentation. In contrast, for much of the prior two decades when mefloquine had been the drug of choice for combat deployments, its use was often undocumented.^2,5^ As many of the subjects in the doxycycline cohort may be expected to have had one or more prior combat deployments, they may also have had prior undocumented exposure to mefloquine. Any lasting effects from such exposure may consequently have been misclassified in the Eick-Cost analysis as being due to doxycycline rather than to mefloquine. Furthermore, the presence of such lasting effects, even if not resulting in a diagnosis but only a prescription of a psychotropic medication to control symptoms, may have made subsequent exposure to mefloquine less likely, as a prior study has shown.^6^ Both this misclassification and bias may have had the effect of decreasing subsequent neurologic and psychiatric diagnoses in the mefloquine cohort relative to the doxycycline cohort, diluting any observed associations. These limitations may potentially be overcome by restricting future investigations to military personnel without prior deployments and without evidence of prior neuropsychiatric contraindications to mefloquine use.^7^

A previous underpowered analysis of U.S. military hospitalizations found a trend toward increased risk with mefloquine of diagnosis of vertigo and adjustment disorder,^8^ a condition which often precedes PTSD. These trends are generally mirrored in the Eick-Cost subgroup analysis. Future investigations should consider a revised outcome of interest defined by informed combinations of these and related neurologic and psychiatric diagnoses. A combination of such diagnoses may be more specific than single diagnoses in retrospectively identifying the characteristic syndrome of lasting neuropsychiatric adverse effects caused by the drug.^9^
